# Dose-dependent consequences of sub-chronic fentanyl exposure on neuron and glial co-cultures

**DOI:** 10.3389/ftox.2022.983415

**Published:** 2022-08-11

**Authors:** Doris Lam, Aimy Sebastian, Chandrakumar Bogguri, Nicholas R. Hum, Alexander Ladd, Jose Cadena, Carlos A. Valdez, Nicholas O. Fischer, Gabriela G. Loots, Heather A. Enright

**Affiliations:** ^1^ Biosciences and Biotechnology Division, Physical and Life Sciences Directorate, Lawrence Livermore National Laboratory, Livermore, CA, United States; ^2^ Computational Engineering Division, Engineering Directorate, Lawrence Livermore National Laboratory, Livermore, CA, United States; ^3^ Nuclear and Chemical Sciences Division, Physical and Life Sciences Directorate, Lawrence Livermore National Laboratory, Livermore, CA, United States

**Keywords:** neuron-glia co-culture, multi-electrode array, cell culture, fentanyl, opioids, neural network

## Abstract

Fentanyl is one of the most common opioid analgesics administered to patients undergoing surgery or for chronic pain management. While the side effects of chronic fentanyl abuse are recognized (e.g., addiction, tolerance, impairment of cognitive functions, and inhibit nociception, arousal, and respiration), it remains poorly understood what and how changes in brain activity from chronic fentanyl use influences the respective behavioral outcome. Here, we examined the functional and molecular changes to cortical neural network activity following sub-chronic exposure to two fentanyl concentrations, a low (0.01 μM) and high (10 μM) dose. Primary rat co-cultures, containing cortical neurons, astrocytes, and oligodendrocyte precursor cells, were seeded in wells on either a 6-well multi-electrode array (MEA, for electrophysiology) or a 96-well tissue culture plate (for serial endpoint bulk RNA sequencing analysis). Once networks matured (at 28 days *in vitro*), co-cultures were treated with 0.01 or 10 μM of fentanyl for 4 days and monitored daily. Only high dose exposure to fentanyl resulted in a decline in features of spiking and bursting activity as early as 30 min post-exposure and sustained for 4 days in cultures. Transcriptomic analysis of the complex cultures after 4 days of fentanyl exposure revealed that both the low and high dose induced gene expression changes involved in synaptic transmission, inflammation, and organization of the extracellular matrix. Collectively, the findings of this *in vitro* study suggest that while neuroadaptive changes to neural network activity at a systems level was detected only at the high dose of fentanyl, transcriptomic changes were also detected at the low dose conditions, suggesting that fentanyl rapidly elicits changes in plasticity.

## Introduction

Fentanyl is one of the most frequently used synthetic opioids in medicine, as an analgesic and for pain management, but its heroin-like euphoric effects have implicated it in an overwhelming number of illicit and lethal drug overdosing cases in the United States. Opioid-related deaths account for two-thirds of all drug-related overdose deaths (Gomes et al., 2018; Ahmad et al., 2021). Fentanyl, like morphine, binds to mu-opioid receptors (MOR) in many brain regions (e.g., ventral tegmental area, prefrontal cortex, amygdala, hippocampus) (Chartoff and Connery, 2014), and this binding event can lead to addiction, tolerance, impairment of cognitive functions, and inhibit nociception, arousal, and respiration as a result of chronic abuse of the synthetic drug (Kosten and George, 2002). The precise mechanisms (e.g., molecular, and functional) of how fentanyl alters neural activity in the brain and leads to these lasting changes in cognitive functions and behavior remain poorly understood.

Recently, brain-on-a-chip (BOC) platforms, which often integrate cell cultures with a multi-electrode arrays (MEA), have emerged as a powerful technology to non-invasively record extracellular action potentials from primary rodent or human induced Pluripotent Stem Cell (iPSC)-derived neurons cultured on these systems (Kamioka et al., 1996; Chiappalone et al., 2003; Halliwell et al., 2021). The ability to monitor the functional dynamics of neural and network activity have been useful in evaluating pharmacological and toxicological effects of compounds on network activity (Chiappalone et al., 2003; Johnstone et al., 2010), and better understand how cellular and molecular changes affect the functional properties of neurons and their networks *in vitro*. More recent work has adopted this MEA technology to assess the natural opioid, morphine (Pollard et al., 2021), and other synthetic opioids, such as methadone (Yao et al., 2020). But no study to date has examined the functional consequences of fentanyl on neural network activity on the MEA technology. There is some evidence from previous studies that indicate a dose-dependent effect of fentanyl (from 0.01 to 10 μM) on neural activity and morphology. Using slice electrophysiology, an increase in hippocampal excitability was observed following 30-minute exposure to fentanyl, while a biphasic neuronal response was observed following sub-chronic exposure to the opioid (e.g., 3 days) wherein neuron cultures had shown dose-dependent changes in dendritic morphology and the expression and clustering of postsynaptic receptors (e.g., AMPA receptors) (Lin et al., 2009; Tian et al., 2015). However, the underlying molecular changes responsible for the functional changes in the activity of neurons and their behavior in a network remain to be elucidated.

In prior work (Enright et al., 2020), we have demonstrated that increased cellular complexity of our rat BOC technology, that includes primary astrocytes and oligodendrocyte precursor cells co-cultured with neurons, is needed to improve the functional relevance of these systems to better mimic the *in vivo* brain, and more accurately reproduce drug responses. We had shown that complex culture systems accelerated the maturation of neural networks, which may have been supported by the concurrent maturation of oligodendrocyte precursor cells to myelin-producing oligodendrocytes, and the reactivity of astrocytes that may contribute to supporting neural network activity. Here, we exposed the same complex neuron-glial cultures to fentanyl doses (0.01 or 10 μM) for 4 days, to mimic a sub-chronic exposure, and monitored electrophysiology from the onset until the last day of exposure. We selected these two concentrations as it was shown in previous studies to induce a dose-dependent or biphasic response (e.g., neural excitability or dendritic morphology and AMPA receptor expression/clustering) from *in vitro* or *ex vivo* systems (Lin et al., 2009; Tian et al., 2015). Bulk RNA sequencing was used to estimate cell abundance in the complex culture and evaluate transcriptomic changes in response to drug challenge. Consistent with prior findings, we identified a dose-dependent effect of fentanyl following sub-chronic exposure, specifically, cultures exposed to the high (but not low) dose showed a decline in their network properties. However, transcriptomic activity was sensitive to fentanyl at both the low and high concentrations, showing gene expression changes to processes involved in synaptic transmission, extracellular matrix, and inflammatory response in the complex neuron-glial cultures.

## Materials and methods

### Cell culture

Complex primary rat cultures containing cortical neurons (Lonza, Walkersville, MD, United States), astrocytes (Lonza), and oligodendrocyte precursor cells (ScienCell, Carlsbad, CA, United States) were prepared as previously described (Enright et al., 2020). Briefly, 6-well Multi-channel system MEA devices (Multichannel Systems, Reutlingen, Germany) or wells in a 96-flat bottom-well plate (Corning) were coated with 0.1 mg/ml poly-D-lysine (Millipore-Sigma) and washed with sterile DI water (4X). Cells were seeded at a density of 3,031 cells/mm^2^ on devices and plates with a seeding percentage of 79% neurons, 16% astrocytes and 5% oligodendrocyte precursor cells, based on previous reports of the brain cells’ ratio during postnatal periods (Bandeira et al., 2009; Clarke and Barres, 2013) and our previous work (Enright et al., 2020). Cultures were maintained in PNGM™ Primary neuron growth medium bulletkit™ (Lonza) in a humidified incubator (37°C, 5% CO_2_). For MEA devices, custom device caps, made from polytetrafluoroethylene (PTFE) housing and a fluorinated ethylene-propylene (FEP) membrane (ALA Scientific, Farmingdale, NY), were used to maintain sterility and to allow for gas exchange. Cultures were fed every 3–4 days with 50% media exchange.

### Chemicals

Concentrated stock of Fentanyl (150 μM, Henry Schein, Melville, NY, United States) was prepared in sterile saline, before diluted with culture media to a 0.15 μM working stock solution for a final concentration of 0.02 μM, or directly diluted to a concentration of 20 μM. At 24 days *in vitro* (DIV), a 50% media exchange for primary cultures was conducted to which the final concentration of fentanyl in the culture well was 0.01 or 10 μM. For the untreated condition, neuron media was added to cultures for the 0 μM treatment condition. Two days after the initial fentanyl exposure, another 50% media exchange was conducted once more with fentanyl or media for the respective treatment conditions. Cultures were exposed to fentanyl for a period of 4 days.

### Electrophysiology recording and data processing

The 6-well format MEA device was placed within a 5% CO_2_-regulated chamber on the heated stage (37°C) of the 256-channel MEA2100 recording system (Multichannel Systems). Recordings started after a 5-minute equilibration time with an action potential spike defined by a lower limit threshold, set at 6.5x the standard deviation of baseline noise, for each electrode. Devices were recorded for 30 min at a sampling frequency of 10 kHz and bandpass filtered between 4 and 4,000 Hz, as before (Novellino et al., 2011; Soscia et al., 2017; Pastore et al., 2018; Lam et al., 2019; Enright et al., 2020; Soscia et al., 2020). Devices were recorded for 30 min twice a week from 7 DIV and onwards to monitor neural and network activity. We have previously shown that neural activity from this complex culture becomes stabilized by 21–32 DIV (Enright et al., 2020). Thus, cultures at 24–26 DIV were exposed to fentanyl and recordings conducted within the first hour of exposure (i.e., 2 × 30 min recordings), and 24, 48, 72, and 96 h after exposure.

### Data analysis, synchrony and network analysis

Time stamped data from each recording was exported as a hdf5 file and analyzed using an in-house custom R package, as in previous studies (Lam et al., 2019; Enright et al., 2020). Data was filtered to remove silent electrodes (<10 spikes) and to classify bursts based on a previously defined burst parameter (Soscia et al., 2017; Lam et al., 2019; Enright et al., 2020; Soscia et al., 2020), and includes: maximum beginning interspike interval (ISI) of 0.1 s, maximum end ISI of 0.2 s, minimum interburst interval (IBI) of 0.5 s, minimum burst duration of 0.05 s, and minimum number of spikes per burst of 6. Feature analyses for spikes and bursts were calculated based on previous work (Charlesworth et al., 2015), and include: firing rate, interspike interval (ISI), burst per minute, burst duration, percentage of spikes within bursts, and interburst interval (IBI). Statistical analysis of spiking and bursting features was determined by calculating the weighted mean and standard deviation over all active electrodes in each array. For fentanyl exposure experiments, the weighted mean and standard deviation (for a specific feature) prior to fentanyl exposure (e.g., baseline) was calculated. Then, the weighted mean for each time point during the sub-chronic fentanyl exposure was calculated and expressed as a fold change relative to baseline activity. Pairwise synchrony between electrodes was calculated as previously described (Lam et al., 2019; Cadena et al., 2020), using SPIKE distance (Kreuz et al., 2013). SPIKE-distance measures the dissimilarity between two spike trains as the average of the *instantaneous* dissimilarity between the two spike trains at different points of the recording. This measure has been previously used for quantifying synchrony in cultured hippocampal neurons (Zapata-Fonseca et al., 2016), understanding social cognition (Eisenman et al., 2015), and to estimate synaptic weights for training robot locomotion (Espinal et al., 2016). As in previous studies (Eisenman et al., 2015; Lam et al., 2019; Enright et al., 2020), spike train distances were subtracted from 1 to obtain a similarity or synchrony measure, such that a value of 1 represents perfect synchrony and a value of 0 denotes complete asynchrony. Additionally, values were normalized by the SPIKE-distance obtained on randomly generated spike trains to compensate for the documented bias of SPIKE distance to assign higher synchrony values to denser spike trains (Sihn and Kim, 2019). Electrodes were separated into communities using the Louvain algorithm for modularity maximization (Blondel et al., 2008). Modularity maximization aims to maximize connectivity strength within communities and minimize connectivity strength between distinct communities. Modularity maximization is used in various applications ranging from identifying excitatory cortical subnetworks (Lee et al., 2016) to detecting communities in social and shopping networks.

### Viability assay

CyQuant™ Lactate Dehydrogenase (LDH) assay (Thermo Fisher Scientific) was performed on the fourth day of exposure to fentanyl, as per vendor’s instructions. Briefly, the supernatant was collected from untreated (0 μM) or fentanyl treated (0.01 or 10 μM) cultures and processed in 96-well plate format, per kit instructions. Absorbances were read at 490 and 680 nm on the Synergy H1 multi-mode microplate reader (BioTek), and absorbance data for the fentanyl treatment normalized to the untreated condition.

### Immunocytochemistry

Primary cultures on the 96-well plate format were fixed with 4% paraformaldehyde (30 min), washed in PBS (5 min, 3X), permeabilized with 0.2% Triton-X100, and then blocked with 10% goat serum in PBS (1 h). Cultures were washed with PBS (3X) before labeling with primary antibodies, prepared in PBS with 3% goat serum, for anti-class III beta-tubulin for neurons (Tuj1, chicken, 1:200, Neuromics, Edina, MN), and anti-Glial fibrillary acidic protein for astrocytes (GFAP, rabbit, 1:500, Sigma-Millipore) and anti-Oligodendrocyte transcription factor for Oligodendrocyte-lineage cells (Olig2, mouse, 1:500, Sigma-Millipore). After primary antibody incubation (4°C, overnight), cells were washed in PBS (3X) and incubated with secondary antibodies prepared in PBS with 3% goat serum (2 h at 30°C). Secondary antibodies (1:200) included: goat anti-mouse linked to Alexa Fluor (AF) 488, goat anti-chicken linked to Alexa Fluor 647 and goat anti-rabbit linked to Alexa Fluor 594 (Life Technologies, Eugene, OR, United States). After secondary antibody incubation, cells were washed with PBS (4X), and incubated with the nuclear stain, DAPI (1:3000, 10 min, ThermoFisher). Imaging of the stained wells at 10X magnification was conducted on the ImageXpress Confocal High-Content Imaging System, (Molecular devices, San Jose, CA, United States), acquiring 6 fields of view.

### Bulk RNA-sequencing, data processing, and analyses

Primary cultures treated and untreated with fentanyl for 4 days were lysed with RLT buffer containing β-mercaptoethanol, and the supernatant collected. Total RNA was extracted and purified from the collected supernatant using the RNAeasy mini spin columns (Qiagen). Sequencing libraries were prepared using Illumina TruSeq RNA Library Preparation Kit v2 (Illumina, San Diego, CA, United States) and sequenced using an Illumina NextSeq 500. Sequencing data quality was checked using FastQC software (https://www.bioinformatics.babraham.ac.uk/projects/fastqc/). Reads were mapped to the rat genome (rn6) using STAR (version 2.6) and read counts per gene were determined using “featureCounts” from Rsubread package (Liao et al., 2014). RUVseq was then used to identify and remove factors of unwanted variation (Risso et al., 2014). Differentially expressed genes were identified using edgeR, controlling for factors of unwanted variation (Robinson et al., 2010). A gene was significantly differentially expressed when its false discovery rate adjusted *p*-value was <0.05 and log2 fold change was >0.5. Gene ontology (GO) and pathway enrichment analysis was performed using ToppGene (Chen et al., 2009) and Enrichr (Kuleshov et al., 2016). Heatmaps were generated using heatmap.2 function in “gplots” R package. Volcano plots were generated using Galaxy Europe (https://usegalaxy.eu/). CIBERSORT, a tool for estimating cell composition of complex tissues from their bulk gene expression profiles, was used to determine the proportion of individual cell types from bulk RNAseq data (Chen et al., 2018). Gene expression signatures from purified populations of neurons, astrocytes, oligodendrocyte precursor cells, newly formed oligodendrocytes, myelinating oligodendrocytes, microglia, and endothelial cells from mouse (Zhang et al., 2014) were used to generate a “signature matrix” which was then used for deconvolution of cell types with CIBERSORT.

### Statistics

Quantified data are expressed as mean ± SEM for the number of replicates indicated. For electrophysiology and LDH data, the statistical significance was analyzed in GraphPad version 9 (GraphPad Software, San Diego, CA, United States) using one-way or mixed model two-way ANOVA followed by Tukey’s post-hoc analysis.

## Results

### Dose-dependent effect on neural network activity during sub-chronic exposure to fentanyl

**FIGURE 1:**
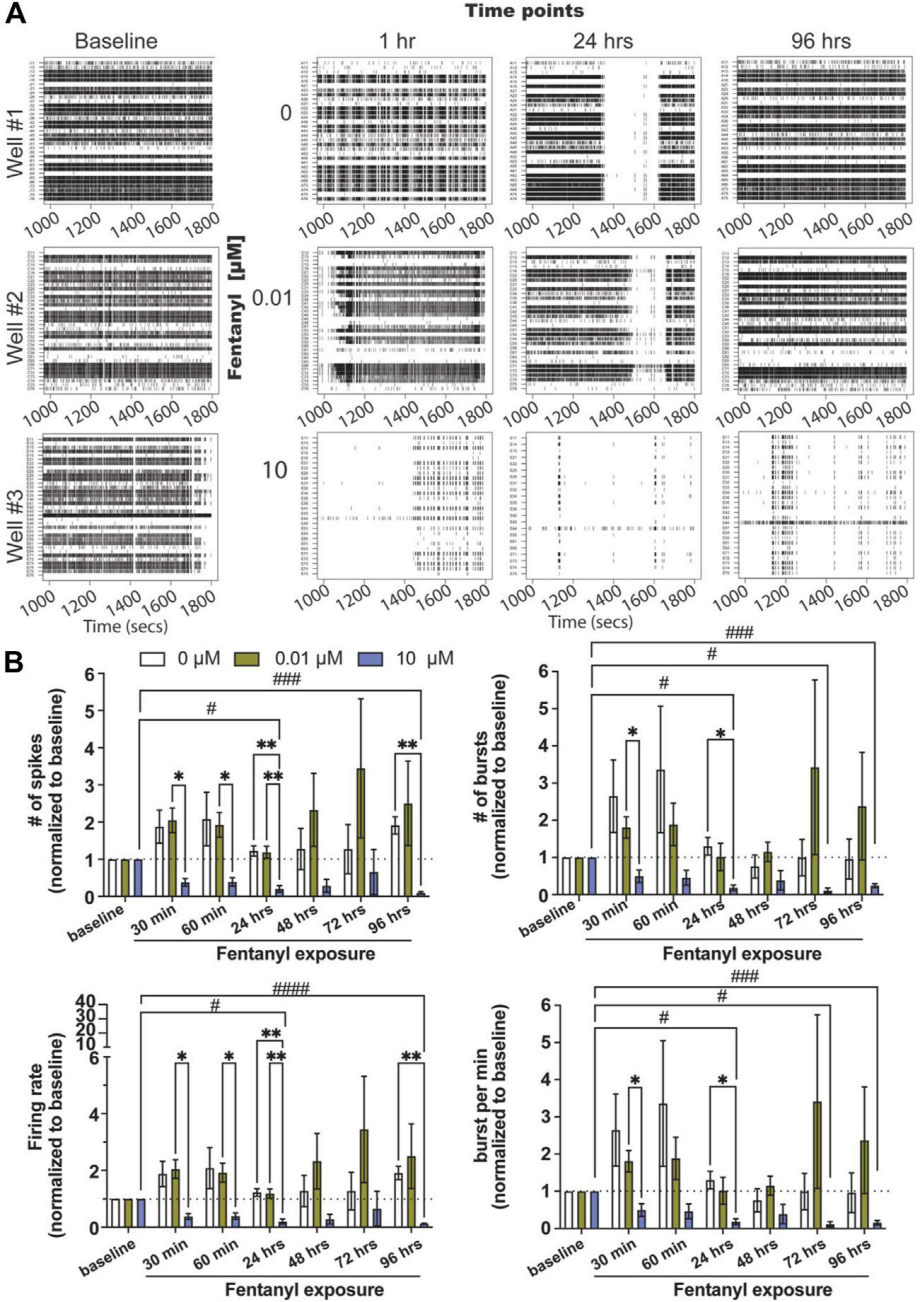
Neural and network activity from complex cultures before and during sub-chronic fentanyl exposure. **(A)** Representative 1800 ms raster plots showing spiking and bursting activity for each electrode (row) before (e.g., baseline) and at 1, 24, and 96 h of exposure to fentanyl at 0, 0.01, and 10 μM. **(B)** Bar graph summarizes the features of spiking (e.g., number of spikes and firing rate, left column) and bursting (number of bursts and burst per minute, right column) before and during sub-chronic fentanyl exposure (*n* = 5–7 wells/treatment condition). Data is normalized to treatment-condition at baseline (dotted line at 1), shown as mean ± SEM, and was analyzed using repeated measures two-way ANOVA with Tukey’s post hoc test. Statistical significances are observed when comparing across treatment conditions (*) and across time points (#) at a level of #*p* < 0.05, ##*p* < 0.01, ###*p* < 0.001, and ####*p* < 0.0001 for the number of symbols indicated.

Complex neuron-glial cultures, with defined seeding percentages of neurons (79%), astrocytes (16%), and oligodendrocyte precursor cells (OPCs, 5%) were selected at random and dosed with either the low or high concentration of fentanyl (e.g., 0, 0.01 and 10 μM) at 24–26 days *in vitro* (DIV) (Figure 1A, baseline). These time points were chosen based on prior work using the BOC technology, where we found neural networks to be mature, displaying both spiking and bursting activity and showing synchronicity between electrodes (Enright et al., 2020). Wells on the BOC were recorded for 30 min within the first hour following dosing (e.g., 30 and 60 min), and daily for a total of 4 days (e.g., 24, 48, 72, 96 h) of exposure. Representative raster plots (Figure 1A) display a dose-dependent effect on spiking and bursting activity within the first hour of exposure, wherein coordinated bursting activity was less apparent with the low dose, 0.01 μM of fentanyl, compared to untreated (e.g., 0 μM) cultures, but a substantial decrease in the number of spiking and bursting activity was observed at the high dose of 10 μM. Daily recordings of the same wells suggested that spiking and bursting activity returned to baseline levels with the low dose treatment condition (Figure 1A; Supplementary Figure S1), while at the high dose of fentanyl it showed reduced levels of spiking and bursting activity that persisted across all time points examined.

Features of spiking (Figure 1B, left column) and bursting activity (Figure 1B, right column) across treatment conditions and time points were quantified, and normalized to baseline activity for each treatment-matched well (=1, dotted line). Normalization to baseline was required as the degree of activity varied from well to well (baseline, Figure 1A). For spiking activity, we did not observe statistical significance differences between the low dose condition across time points or when compared to the untreated condition (*n* = 5–7 wells/treatment condition) for the number of spikes, firing rate, and interspike interval (Supplementary Figure S2A). However, the high dose condition had shown a significant decrease in most features of spiking activity (but not interspike interval, Supplementary Figure S2A) as early as 30 min, and persisted at 24 and 96 h when compared to untreated or the low dose condition. A similar effect was observed with bursting activity for the low and high doses of fentanyl. For the low dose of fentanyl, no statistically significant differences were observed when compared to untreated condition or across time points for the number of bursts and bursts per minute (Figure 1B), burst duration (Supplementary Figure S2B) and interburst interval (Supplementary Figure S2B). However, for the high dose of fentanyl, a statistically significant decrease in activity, below baseline level of activity, was observed when compared to the untreated condition and the low dose or across time, as early as 30 min, and persisted at 24, 72, and 96 h for the number of bursts and burst per minute. For burst duration, a significant decrease was observed only at the 96 h time point, and no effect was observed for the interburst interval (Supplementary Figure S2B).

**FIGURE 2:**
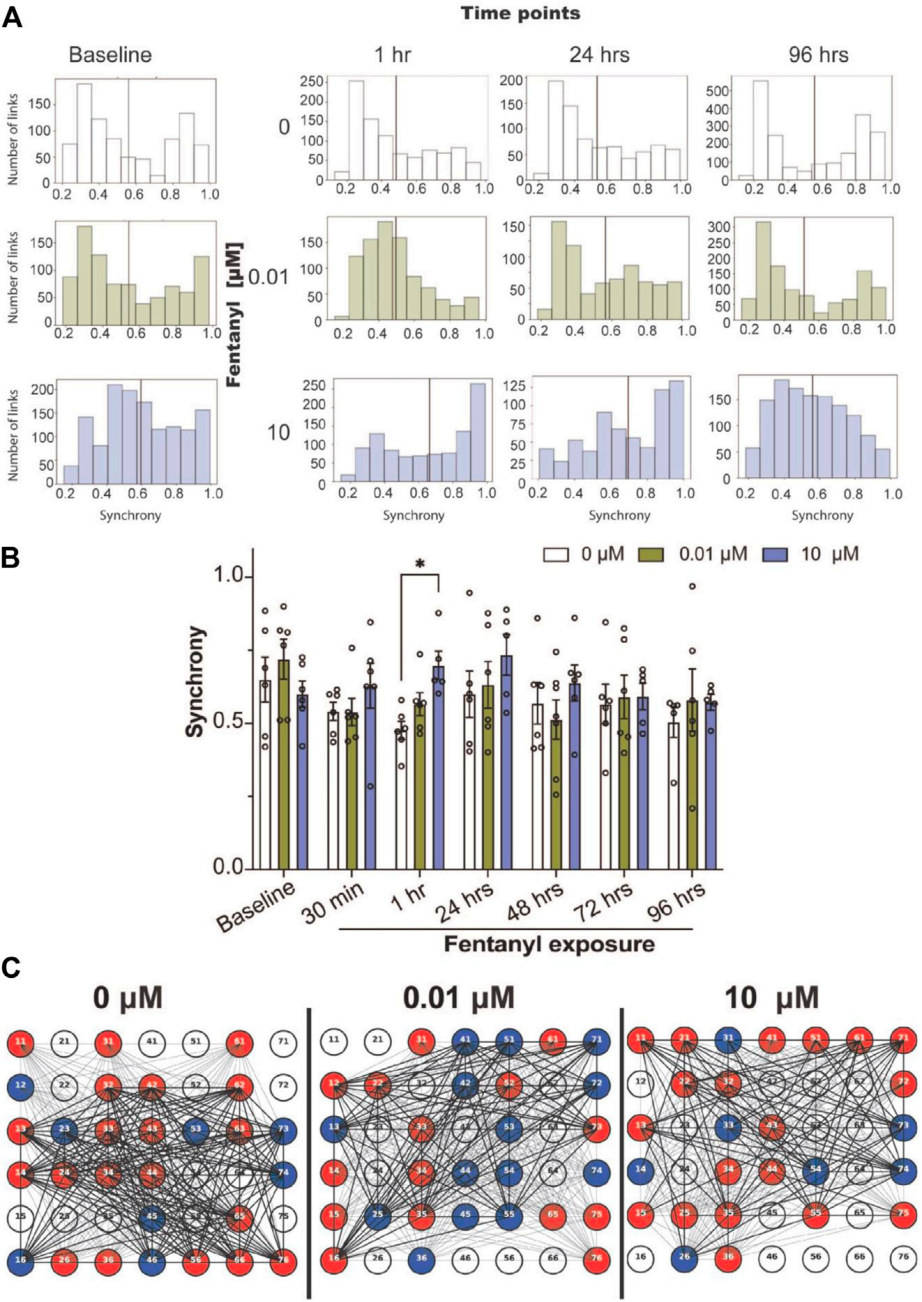
Synchrony and community analysis of neural network activity before and during sub-chronic fentanyl exposure. **(A)** Histogram summarizes the distribution of the degrees of synchronized networks detected from all networks (or links) pooled from BOC devices in the untreated- (e.g., 0 μM) or fentanyl-treated (e.g., 0.01 and 10 μM) conditions (*n* = 5–7 wells/treatment condition), shown at specific time points over the course of 4 days. Black line indicates the mean synchrony value of all detected networks within the treatment condition. **(B)** Bar graph summarizes the average synchrony value from each well within the BOC device across the 7 time points. **(C)** Representative well on the BOC device for complex cultures at the 96 h time point that were untreated or treated with 0.01 or 10 μM of fentanyl. Electrodes are numbered and are shown as inactive (white), or active and part of a community (color). Networks with a synchrony value >0.7 is shown with a thick black line and values <0.7 are shown as a thin black line. Data is shown as mean ± SEM for the number of wells in each treatment condition (*n* = 5–7 wells) and was analyzed using the mixed-model two-way ANOVA with Tukey’s post hoc test. Statistical significance was observed between treatment condition at a level of *p < 0.05.

To quantify the degree of synchrony for each network or the connectivity between a pair of electrodes, we used the SPIKE-distance approach (Kreuz et al., 2013; Eisenman et al., 2015; Cadena et al., 2020; Enright et al., 2020), which computes a SPIKE-distance between every pair of active electrodes for each BOC device, where synchrony values close to 1 denote high degree of synchrony, and values close to 0 denote asynchrony. We examined the distribution of synchrony scores across all networks (or links) detected from the cultures treated with and without fentanyl (pooled from *n* = 5–7 cultures per treatment condition, Figure 2A). We found that prior to treatment, synchronized networks display a bimodal distribution, containing a population of networks that cluster around synchrony values of ∼0.3–0.5 and ∼0.9–1.0. Within an hour of treatment, we observed a shift in the distribution of synchrony values to a unimodal distribution. Especially, networks from 0 to 0.01 μM treated cultures showed a leftward shift favoring the low synchrony values (∼0.3–0.5). Meanwhile, networks from the 10 μM treated cultures showed a rightward shift, favoring high synchrony values (∼0.9–1.0). This behavior was also observed at 24 h. However, at 96 h, the 0.01 μM treated cultures reverted to the bimodal distribution. When we examined the average synchrony value from all networks in a device for each treatment condition across time points, at a device level (Figure 2B), statistical significance was only observed at the 1-hour time point, in which the high dose condition had shown a greater average synchrony value compared to the untreated condition. Thus, it appears that by 96 h of exposure to fentanyl, the synchronization of networks returns to levels similar to baseline, and was not significantly different to untreated cultures, as shown in the representative BOC device with inactive (white) and active electrodes (color) and the functional links (or networks, black lines) detected (Figure 2C).

### Dose-dependent effect on cell viability

**FIGURE 3:**
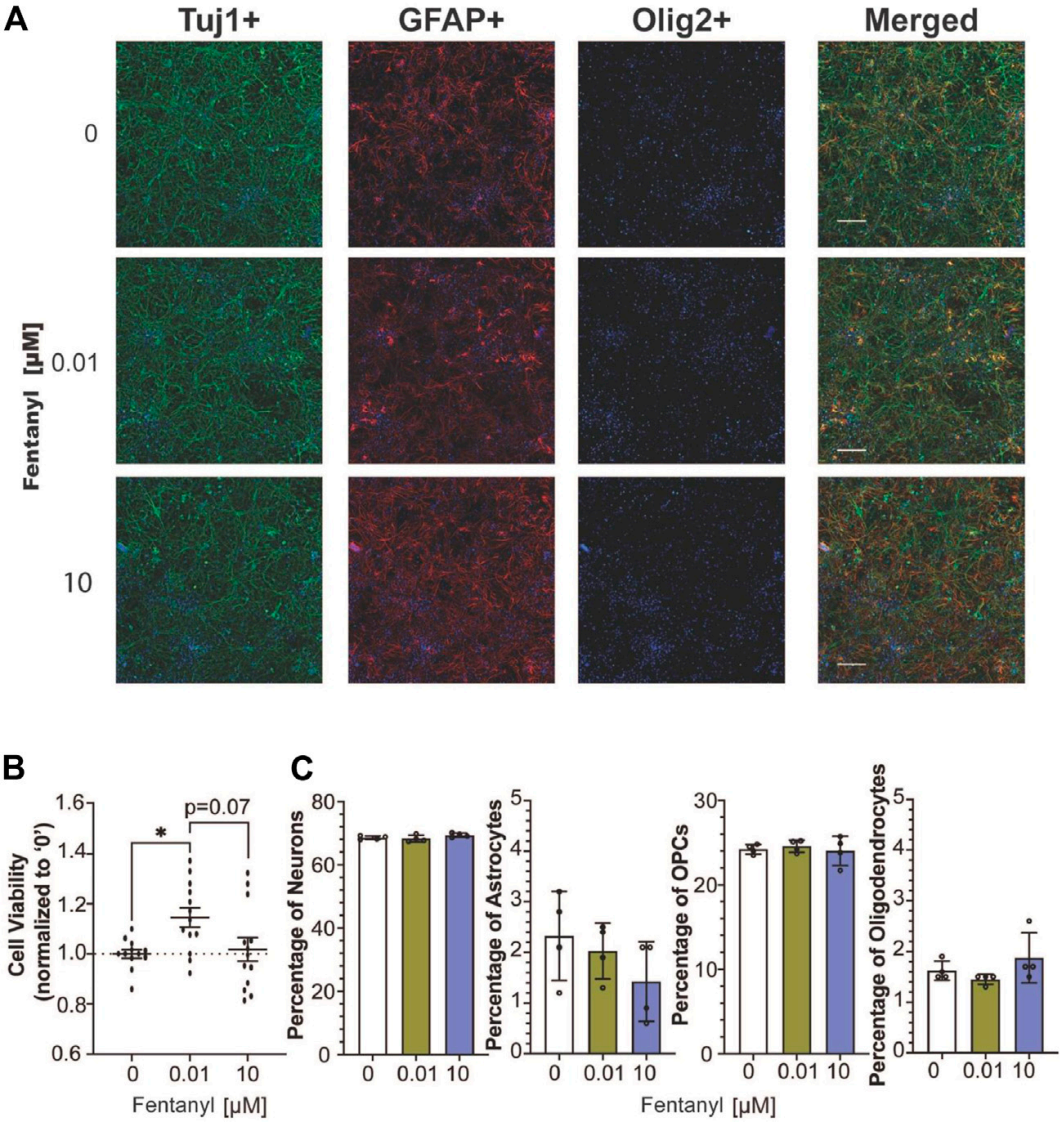
Cell viability of complex culture containing neurons, astrocytes, and oligodendrocyte precursor cells after 96 h of exposure to fentanyl. **(A)** Representative images of Tuj1-, GFAP-, Olig2- stained cells co-stained with the nuclear marker, DAPI (blue), and merged image of all three cellular markers. **(B)** Scatter plot summarizes the amount of LDH released in the supernatant of fentanyl-treated cultures (*n* = 4 biological replicates with 3 technical replicates) normalized to the untreated (control) condition (e.g., 0 μM, dotted line). **(C)** Bar graph summarizes the estimated percentage of neurons, astrocytes, OPCs, and oligodendrocytes in the complex culture nuclear count (*n* = 4 biological replicates). Data is shown as mean ± SEM and was analyzed using one-way ANOVA with Tukey’s post hoc test. Statistical significances are observed at a level of *p < 0.05.

Following 4 days of fentanyl exposure, we observed no drastic changes in the complex cell population, using immunocytochemistry to identify neurons (e.g., Tuj1-positive cells), astrocyte (e.g., GFAP-positive cells), and oligodendrocyte-lineage cells, including immature oligodendrocyte precursor cells and mature myelin-producing oligodendrocytes (e.g., Olig2-positive cells) (Pepper et al., 2018) population (Figure 3A). To evaluate whether the observed changes in activity were attributed to neuronal health, we examined cell viability by quantifying LDH released from cultures after 96 h of fentanyl exposure (Figure 3B). LDH is a cytoplasmic enzyme that is released from cells with compromised membrane integrity, a feature of cells undergoing cell death (e.g., apoptosis, necrosis). We determine that only the cultures treated with the low dose of fentanyl displayed a significant (1.15-fold) increase in LDH levels, relative to the untreated controls. This trend was also observed when taking the cell count of DAPI-positive cells from immunocytochemistry data (Figure 3A) wherein a significant (*p* < 0.05) increase in the number of DAPI positive cells (mean ± SEM cells per field of view) within the low dose condition (2168 ± 69 cells) compared to the untreated (1549 ± 97 cells) and high dose (1840 ± 143) conditions (*n* = 3 wells/treatment conditions). Also, when we estimated the abundance of each cell type within the complex cell culture condition using CIBERSORT, an analytical tool to extrapolate the abundance of each cell type within a mixed cell population based on bulk RNA sequencing data (Figure 3C), no statistically significant differences were observed across treatment conditions for the estimated percentage of the neuron, astrocyte, OPC, and oligodendrocyte population (*n* = 4 cultures/treatment condition), suggesting that fentanyl exposure is not selectively toxic to neurons or any specific glial cell population. Thus, while LDH levels had risen in only the low dose condition, it may be likely attributed to compromised membrane integrity without affecting cell viability.

### Dose-specific changes in gene expression follow sub-chronic fentanyl exposure

**FIGURE 4:**
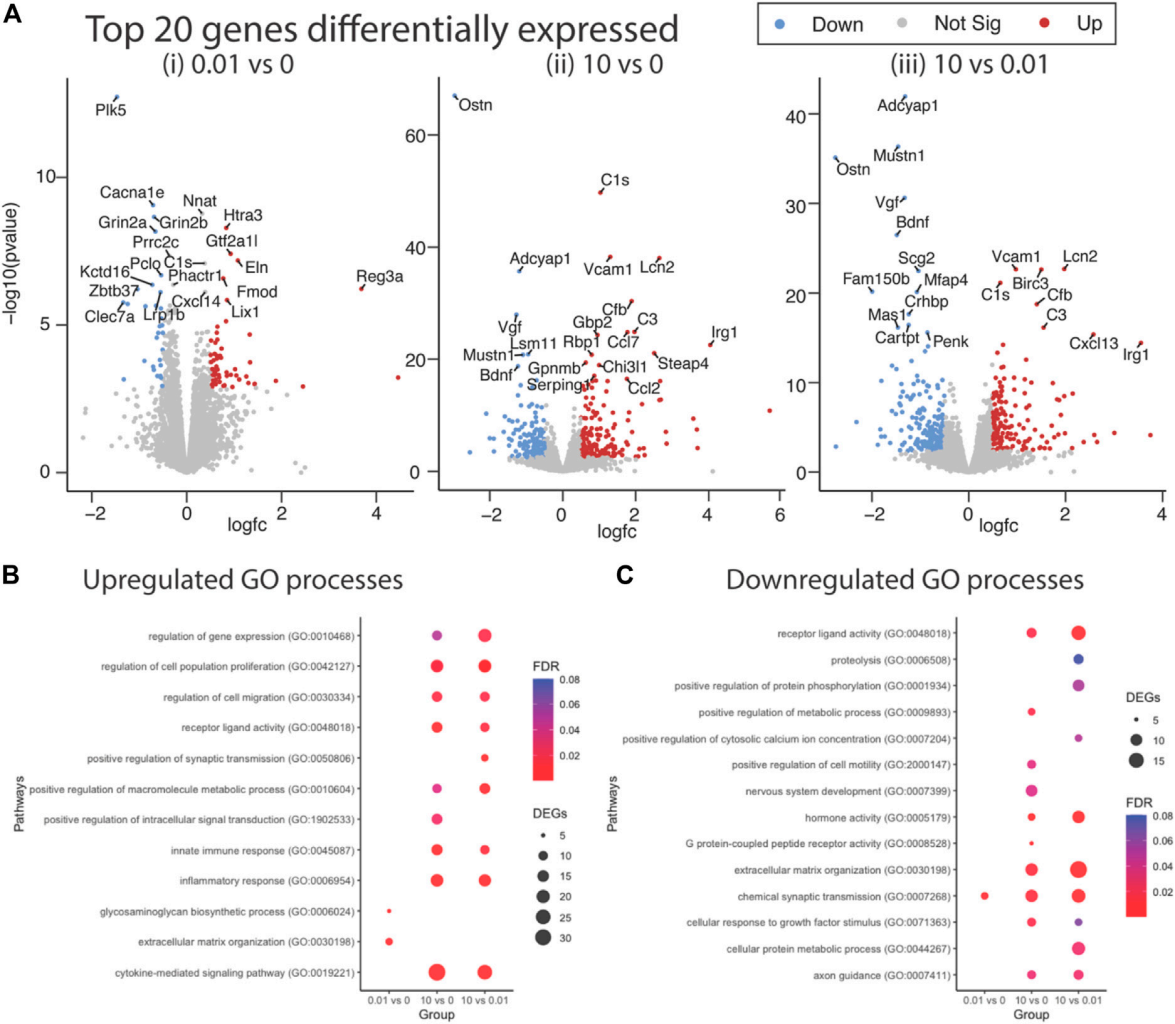
Differential gene expression in complex culture following sub-chronic exposure to low versus a high dose of fentanyl. **(A)** Volcano plots of statistical significance versus the magnitude of gene expression between treatment conditions: 0.01 vs. 0 μM fentanyl (i), 10 vs. 0 μM of fentanyl (ii), and 10 vs. 0.01 μM (iii) of fentanyl. Top 20 differentially expressed genes (DEGs) are highlighted. Dot plots showing enriched biological process associated with upregulated **(B)** and downregulated **(C)** genes. Circle size represents the number of genes in each Gene Ontology (GO) category and the color indicates the false discovery rate associated with each GO category.

**FIGURE 5:**
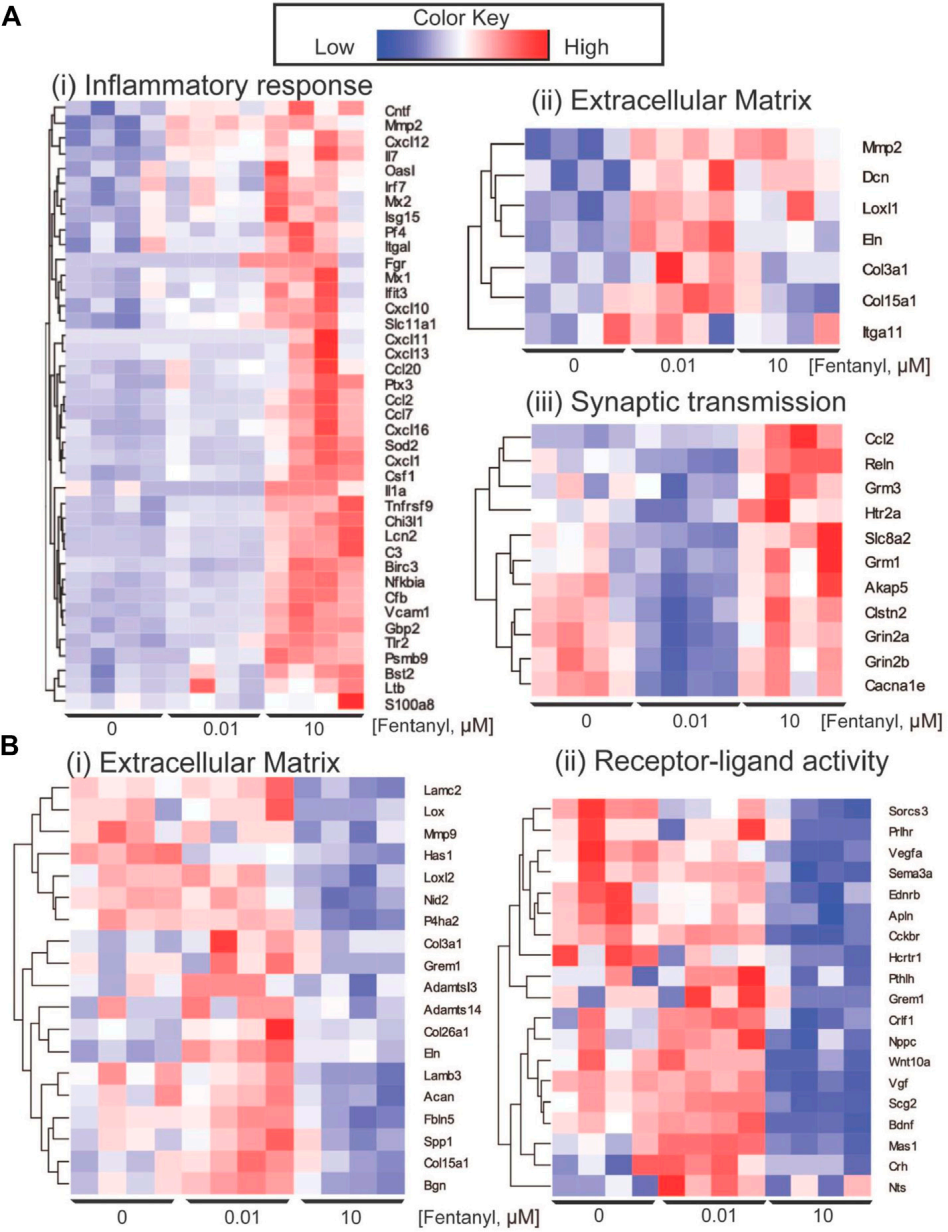
Key differentially expressed genes. **(A)** Heatmap of genes showing the upregulation of genes in complex culture conditions exposed to 10 μM of fentanyl for GO biological processes involved in inflammation response (i) and synaptic transmission (iii), and specific to 0.01 μM of fentanyl for processes involved with the extracellular matrix. **(B)** Downregulation of genes in complex cultures exposed to 10 μM of fentanyl for processes involved in the extracellular matrix (i) and receptor-ligand activity (ii).

Differential gene expression (DEG) analysis identified 87 genes differentially expressed between low dose vs. untreated, 318 genes between high dose vs. untreated and 352 genes between high vs. low dose (DEGs; abs (log_2_FC) > 0.5 and FDR < 0.05) (Figure 4A). Ontology enrichment analysis of DEGs identified biological processes associated with the extracellular matrix, specifically glycosaminoglycan biosynthetic processes and extracellular matrix organization as uniquely enriched in the low dose of fentanyl cultures compared to the untreated condition (Figure 4B), while genes involved with chemical synaptic transmission were downregulated (Figure 4C). DEGs upregulated in the high dose of fentanyl cultures compared to the untreated condition were associated with receptor-ligand activity and downstream intracellular signal transduction, processes involved in transcription and translation processes (e.g., regulation of gene expression, metabolic processes) and inflammation (e.g., innate immune response, cytokine-mediated signaling pathway, proliferation, and migration). Biological processes associated with downregulated DEGs in the high dose versus untreated condition also included receptor-ligand activity (e.g., G-protein coupled peptide receptor activity) and downstream intracellular signal transduction (e.g., protein phosphorylation, regulation of metabolic processes,) and several cellular functions (e.g., cell motility, extracellular matrix organization axon guidance, nervous system development, response to growth factor stimulus, chemical synaptic transmission). Ontology enrichment analysis revealed that there were unique biological processes specific to the high dose condition and low dose condition. We next examined genes belonging to selected biological processes, in particular we focused on upregulated genes associated with inflammatory responses, extracellular matrix and synaptic transmission (Figure 5A) and downregulated genes associated with the extracellular matrix and receptor-ligand activity (Figure 5B). For inflammation, high dose of fentanyl condition was enriched with genes involved with acute inflammatory response (e.g., *C3, Il1a, Lcn2, S100a8, Vcam1),* chemokine-mediated signaling (e.g., *Ccl2, Ccl20, Ccl7, Cxcl10, Cxcl11, Cxcl12, Cxcl13, Pf4*), phagocytosis (*C3, Ccl2, Fgr, Ptx3, Slc11a1, Tlr2*), negative regulation of viral process (e.g., *Bst2, Isg15, Mx1, Oasl, Ptx3*). Genes for the ionotropic glutamate receptor (e.g., *Grin2a, Grin2b*) and those relevant to calcium ion transport (e.g., *Cacna1e* and *Slc8a2*) were enriched in the high dose and untreated conditions, but interestingly downregulated in the low dose condition. Interestingly, genes relevant to the metabotropic glutamatergic receptors and signaling pathways (e.g., *Grim1*, and *Grim3*, and *Akap5)* were upregulated in only the high dose condition and not low dose or untreated conditions. Lastly, several genes involved in the extracellular matrix were upregulated in low dose condition, specifically genes that have been previously associated with invasion and metastasis (e.g., C*ol3a1, Itga11, Mmp2*) and collagen fibril organization (e.g., *Col3a1, Loxl1, Col15a1*) (Figure 5A).

We also observed a downregulation of a number of genes associated with the extracellular matrix downregulated in the high dose condition. These included genes involved in collagen fibril organization and assembly (*Adamts14, Col3a1, Grem1, Lox, Loxl2, Mmp9*), and extracellular matrix assembly (e.g., *Has1, Lamb3*). In addition, genes involved with receptor-ligand activity, specifically neuropeptide signaling pathway (e.g., *Hcrtr1, Nts, Prlhr, Sorcs3*), negative regulation of cell development (e.g., *Ednrb, Nppc, Pthlh, Sema3a, Vegfa*), GPCR (e.g., *Cckbr, Ednrb, Hcrtr1, Mas1, Prlhr)* were also downregulated in high dose condition.

## Discussion

Much of our understanding of chronic fentanyl use has been well documented in behavioral studies of animal models (Han et al., 2022). However, how chronic fentanyl exposure regulates brain activity that leads to changes in behavior remains poorly understood, particularly in *in vitro* and *in vivo* models. Previous *in vitro* and *ex vivo* studies have evaluated the effects of fentanyl on network activity (using slice electrophysiology), spine morphology of neurons, and receptor expression (e.g., AMPA receptors) at doses considered low (e.g., 0.01, 0.1 μM) and high (10 μM) and identified a dose-dependent or biphasic response from *in vitro* or *ex vivo* systems. (Lin et al., 2009; Tian et al., 2015). The exposure period for these studies varied (e.g., 3 days vs. 30 min). In the present study, we leveraged the BOC technology to provide a time series data set over a period of time that can be age-matched to previous studies, as shown here at 30 min, 1 h, and 24, 48, 72, and 96 h in which we monitored neural and network activity over the course of a 4-day exposure period (Figure 1), to better understand how the length and dose of fentanyl exposure affects network activity in the brain.

While the concentration used in the present study was determined from previous *in vitro* or *ex vivo* systems, (Lin et al., 2009; Tian et al., 2015), it is unclear whether the concentration is relevant to fentanyl levels in the brain of chronically exposed rodents. Nevertheless, electrophysiology data suggests that a high (but not low) dose of fentanyl, 10 μM, had a significant effect on reducing neural network activity, affecting features of spiking (e.g., number of spikes, firing rate) and bursting (e.g., number of bursts, burst per minute), as early as 30 min of exposure and sustained for up to 96 h of exposure. Previous reports have suggested that concentrations greater than 10 μM is likely to have off-target effects, for example inhibition of voltage-gated sodium channels (Isensee et al., 2017), altering intracellular calcium kinetics (McDowell, 2003), possibly attributed to inhibition of voltage-gated Ca^2+^ channels (N-, P-, Q-type) (Schroeder et al., 1991; Moises et al., 1994; Rusin and Moises, 1995), α1A and α1B adrenoceptor subtypes (Torralva et al., 2020), dopamine D4.4 and D1 receptor subtypes (Torralva et al., 2020), which may contribute to reduced network activity observed in the present study. Although, cultures exposed to the low dose, 0.01 μM, of fentanyl were previously shown to reduce presynaptic inputs (e.g., collapse of dendritic spines and decreased AMPA receptor clusters) (Tian et al., 2015), network activity was comparable to the untreated cultures in the present study, despite showing a slight increase in the level of spiking and bursting activity. Interestingly, when we examined the dynamics of synchronized networks within the treatment conditions (Figure 2), the distribution of synchrony values shifted, with most networks displaying a low degree of synchronization for the untreated and low dose condition, which may be attributed to mechanical disturbance of dosing the culture since the untreated condition also displayed the same shift. The observation that a high proportion of networks in the high dose condition (at 1 and 24 h time points) displayed a high degree of synchronization may be to accommodate the reduced level of spiking and bursting activity. While the distribution of synchrony values returned to baseline level by 96 h for the untreated and low dose condition, networks in the high dose condition showed a broader range of synchrony values. At a device level, the average synchrony values of all active networks had shown that there was no difference between treatment condition by 96 h, despite notable differences in the distribution of synchrony values. It is unclear whether the shift in the degree of synchrony across networks is a beneficial or harmful effect of opioid within the complex culture system. Specifically, is the shift from bimodal to unimodal distribution and the strength of synchronicity between neurons in a network a compensation mechanism for the change in spiking or bursting activity observed in the high dose condition? While the interpretation of the dynamics of synchronized networks is limited in this *in vitro* system, it would be interesting to see if a similar phenomenon (using *in vivo* electrophysiology) was observed in an animal model exposed to fentanyl.

Cell viability (e.g., LDH assay, Figure 3B) or the relative proportion of neurons (identified using CIBERSORT, Figure 3C) did not appear to affect the observed changes in network activity. In particular, after 4-days of exposure to fentanyl, levels of the LDH enzyme in the culture supernatant, a measure of membrane integrity and/or indirectly cell death, in the high dose condition was comparable to untreated cultures, and similarly the total number of cells and estimated proportion of cells (e.g., neurons, astrocytes, and oligodendrocyte precursor cells or oligodendrocyte) from age-matched treated cultures was also comparable to untreated cultures. The doses used in the present study did not appear to induce cytotoxicity at the 4-day time point, as expected, since >250 μM of fentanyl has been previously shown to induce toxicity in neurons after 24 h of exposure (Sogos et al., 2021).

Interestingly, transcriptomic analysis revealed gene expression changes in complex cultures after 4-days of exposure to fentanyl, regardless of the low versus high dose of the opioid (Figure 4A). Notably, gene expression changes in the low dose condition were specific to processes involved with the extracellular matrix (ECM, e.g., glycosaminoglycan biosynthetic process, or ECM organization), and only a marginal effect on genes involved in chemical synaptic transmission (Figures 4B,C, 5Aii,iii). Thus, the change in genes involved in synaptic transmission was not sufficient to alter network activity as observed on the BOC technology. More pronounced gene expression changes occurred at the high dose condition, specifically an upregulation of genes involved in inflammation and synaptic transmission (Figures 5Ai,iii). However, there were some cases where gene expression changes were no different to untreated levels, despite an increase in the same gene under the low dose condition. It is possible that there is a compensation mechanism in the high dose condition to regulate the transcriptome back to baseline levels, specifically for processes involved in the extracellular matrix and receptor-ligand activity (Figure 5B). However, future work is needed to elucidate whether specific receptor-ligand expression (e.g., extracellular matrix-specific and ionotropic or metabotropic receptors), and its corresponding signaling pathways have been amplified or suppressed, as a mechanism for this transcriptomic compensation observed in the high dose condition. To our knowledge, transcriptomic analysis of neuronal and/or glial cultures and in animal models following fentanyl exposure remains to be determined. While transcriptomic analysis of the nucleus accumbens or striatum of animal models exposed to other opioids, such as morphine and heroin, have been informative in understanding the effects of opioids in the brain (Korostynski et al., 2007; Piechota et al., 2010; Salas et al., 2011; Imperio et al., 2016; Avey et al., 2018; Martin et al., 2018; Torralva et al., 2020), more studies are needed to identify the specific consequences of fentanyl in the rodent brain, and whether the dose-dependent effects observed *in vitro,* in the present study, mirror the effect *in vivo*.

## Data Availability

The datasets presented in this study can be found in online repositories. The names of the repository/repositories and accession number(s) can be found below: NCBI Gene Expression Omnibus (GEO), GSE207606
