# Supplementary material for: Dose-dependent consequences of sub-chronic fentanyl exposure on neuron and glial co-cultures
Source: Front Toxicol. 2022 Aug 11;4:983415. doi: 10.3389/ftox.2022.983415 (PMC9403314; doi:10.3389/ftox.2022.983415)
Supplement: Supplementary file 1 [file DataSheet1.docx]

Supplementary Material

## Supplementary Figures


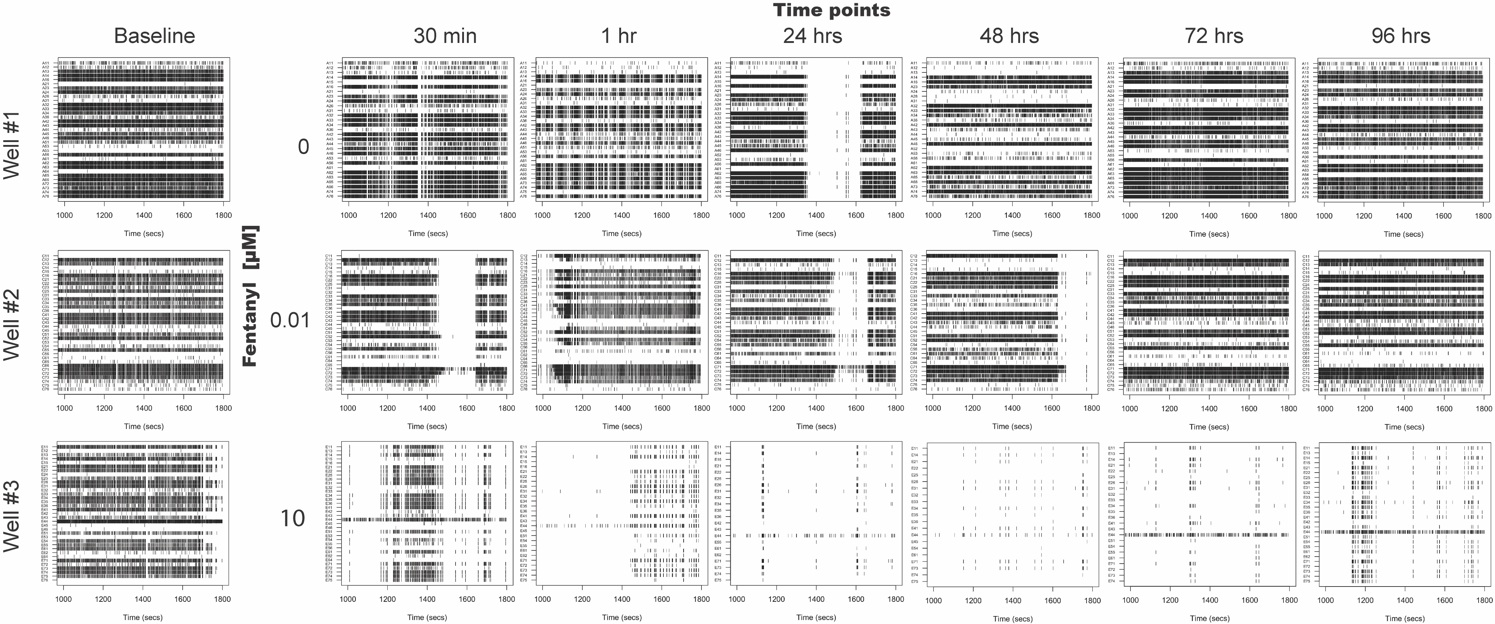


Supplementary Figure 1. **Spike trains from complex cultures before and during sub-chronic fentanyl exposure.** Representative 1800 ms raster plots showing spiking and bursting activity before (e.g., baseline) and at 30 min, 1 hour, and 24, 48, 72 and 96 hours of exposure to fentanyl at 0, 0.01, and 10 µM.


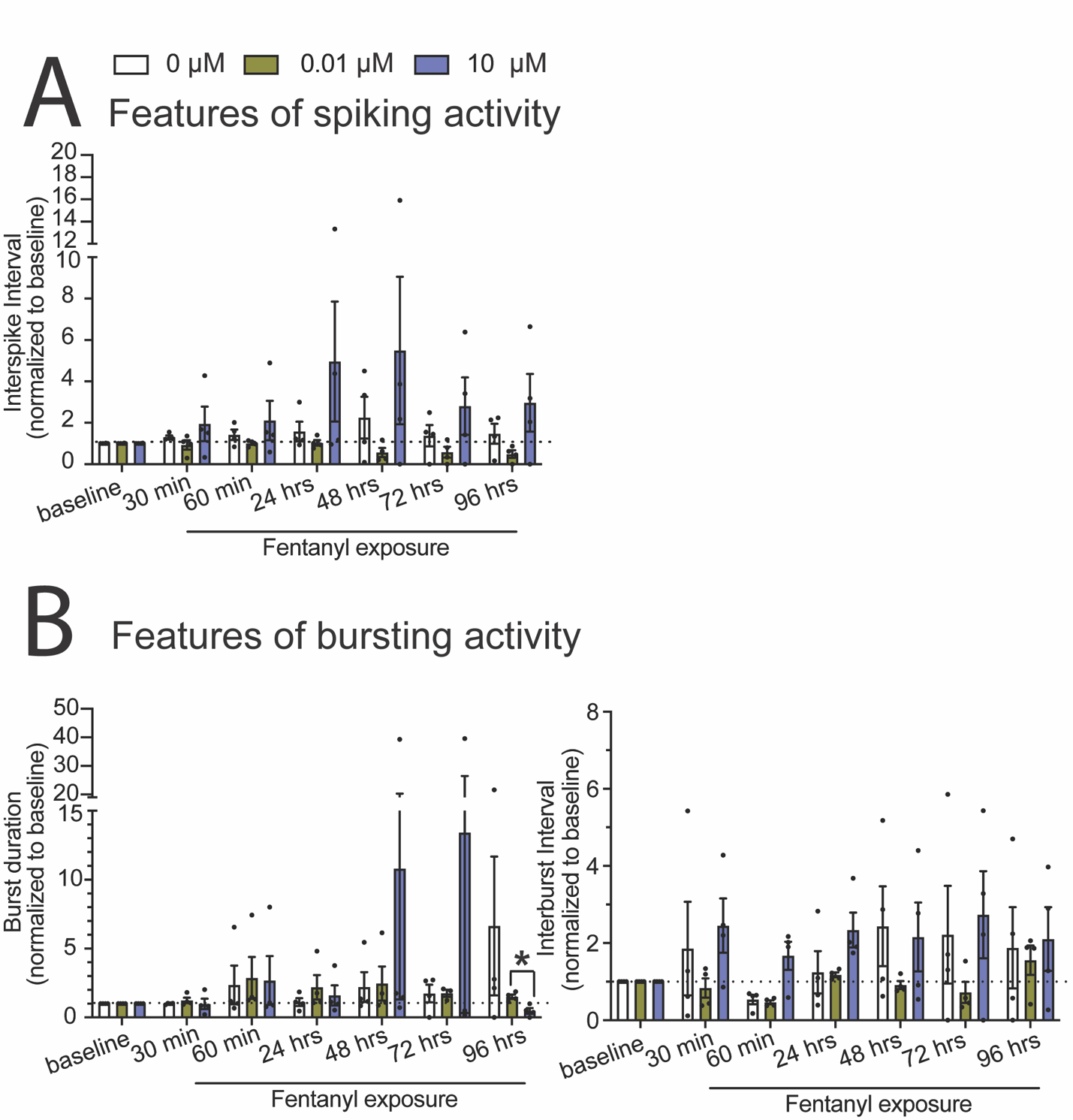


Supplementary Figure 2. **Additional features of** **neural and network activity from complex cultures before and during sub-chronic fentanyl exposure.** Bar graph summarizes additional features of spiking (e.g., interspike interval, **A**) and bursting (e.g., burst duration, and interburst interval, **B**) (n=5-7 wells/ treatment condition). Data is normalized to treatment-condition at baseline (dotted line at 1) and is shown as mean ± SEM and was analyzed using repeated measures two-way ANOVA with Tukey’s post hoc test. Statistical significances is observed at a level of *p<0.05.
